# Perceptions, facilitators, and barriers of participation for a behavioral weight loss group-based telehealth program for breast cancer survivors: a qualitative study

**DOI:** 10.1007/s00520-024-08999-x

**Published:** 2024-11-20

**Authors:** Yangzi Liu, Elizabeth De Jesus, Macy Goldbach, Robert S. Krouse, Carmen E. Guerra, Katharine A. Rendle, Tamara J. Cadet, Kelly C. Allison, Julia Tchou

**Affiliations:** 1 Department of Surgery, Perelman School of Medicine, University of Pennsylvania, Philadelphia, PA 19104 USA; 2 Department of Surgery, Cpl. Michael J. Crescenz Veterans Affairs Medical Center, Philadelphia, PA USA; 3 Department of Medicine, Perelman School of Medicine, University of Pennsylvania, Philadelphia, PA USA; 4 Abramson Cancer Center, University of Pennsylvania, Philadelphia, PA USA; 5 Department of Family Medicine & Community Health, Perelman School of Medicine, University of Pennsylvania, Philadelphia, PA USA; 6 School of Social Policy & Practice, University of Pennsylvania, Philadelphia, PA USA; 7 Department of Psychiatry, Perelman School of Medicine, University of Pennsylvania, Philadelphia, PA USA

**Keywords:** Breast cancer; Weight loss; Obesity; Healthcare disparity

## Abstract

**Purpose:**

Results from the pilot *G*roup-bas*E*d *T*elehealth behavioral *We*ight *L*oss (GET-WEL) Program (NCT04855552) showed that fewer Black breast cancer survivors (BCS) enrolled than White BCS. Black participants also lost less weight than White participants. Little is known about mitigating factors or how best to implement such programs equitably. In this study, we explored facilitators and barriers in Black and White BCS who did or did not participate in GET-WEL.

**Methods:**

BCS who are overweight or obese (body mass index (BMI) ≥ 25 kg/m^2^) and who had previously been assessed for their willingness to participate in GET-WEL were invited to participate in a semi-structured telephone interview conducted from June to August 2023. Interviewees were purposefully sampled from those who did (participants) and did not (non-participants) enroll in GET-WEL. Interviews were coded and analyzed via comparative thematic analysis.

**Results:**

Of the 24 interviewees, 9 (8 White, 1 Black) were GET-WEL participants, and 15 (8 White, 6 Black, 1 Asian) were non-participants. There were no thematic differences between Black and White BCS. Most non-participants lacked awareness that the Program was recruiting. Program accountability, session flexibility, and pre-existing exercise routines emerged as facilitators while inability to identify enjoyable physical activities, difficulty accessing healthy foods, and competing work/life priorities emerged as barriers.

**Conclusion:**

Our results suggest that enhancing Program awareness and outreach may increase enrollment in minoritized BCS. Resources providing healthy foods and support to ease competing work/life priorities may help BCS maintain healthy lifestyles during and after GET-WEL. These results may help inform future large-scale GET-WEL implementation.

## Introduction

Obesity, defined as BMI > 30 kg/m^2^, is associated with increased incidence and worse overall outcomes and cancer-specific survival in breast cancer patients as compared to breast cancer patients without obesity [1, 2]. Breast cancer survivors (BCS) with obesity have an increased risk of total and breast cancer-specific mortality and developing a second primary breast cancer or contralateral breast cancer as compared to BCS without obesity [1, 3].

Weight loss has been associated with a decreased risk of breast cancer recurrence in survivors [4, 5] and other chronic conditions such as type 2 diabetes and cardiovascular disease. As such, intentional weight loss programs have been created specifically for BCS to improve health and breast cancer outcomes [4, 6, 7]. Prior studies have shown that weight loss programs are effective in improving anthropometric outcomes (weight, BMI, body composition) in BCS [8]. However, there exists an observed difference in total weight loss between women of different races, namely non-Hispanic White participants having greater rates of clinically meaningful weight loss [6] compared to Black women. We recently conducted a *G*roup-bas*E*d *T*elehealth behavioral *We*ight *L*oss (GET-WEL) Program for BCS with obesity and found lower recruitment and retention of Black BCS. For those who enrolled in GET-WEL, Black BCS lost less weight compared to White BCS.

Reasons for lower recruitment and barriers to retention of Black BCS in the context of a behavioral weight loss program such as our pilot GET-WEL Program are not well understood. In this qualitative study, we aim to explore facilitators and barriers in Black and White BCS who did or did not participate in GET-WEL to gain insight into factors that may contribute to the lower enrollment rate of Black BCS.

## Methods

### Interviewees

Interviewees for this study were drawn from those who have previously completed health survey questionnaires [9] to assess their willingness to participate in our pilot GET-WEL Program (NCT 04855552) and who, subsequently, did (participants) or did not (non-participants) participate in GET-WEL Program. Inclusion criteria included a diagnosis of breast cancer, aged 18 years or older, Eastern Cooperative Oncology Group (ECOG) performance of 0 or 1, BMI of ≥ 25 kg/m^2^, completion of adjuvant radio- and/or chemotherapy for breast cancer at least 6 months prior to recruitment, and free of any other current cancer diagnosis. Those who were currently using weight-loss medication (over the counter or prescription), currently participating in a behavioral weight loss program, self-reporting alcohol or substance abuse within the past 12 months, including at-risk drinking (current consumption of more than 14 alcoholic drinks per week), history of anorexia nervosa or bulimia nervosa, inability to provide informed consent, current pregnancy, non-English speaking, uncontrolled medical conditions, and steroid use or use of other medications known to cause weight gain were excluded from the study.

Questions were specifically designed for two groups of interviewees stratified by those who did (participants) and those who did not participate (non-participant) in GET-WEL. We invited BCS to participate in these semi-structured interviews via purposive sampling with a goal of interviewing Black and White BCS in a 1:1 ratio. This research was approved by the University of Pennsylvania IRB and done in accordance with the ethical standards as laid down in the 1964 Declaration of Helsinki.

### The GET-WEL Program

The GET-WEL Program was a 20-week program adapted from the Diabetes Prevention Program, [10] a well-validated program used previously for individuals with many presenting health issues, including cancer survivors [11, 12]. The Program was led by a psychologist trained in behavioral weight loss and sessions addressed domains associated with behavioral weight managements such as nutrition, exercise, stress and emotion management, and lifestyle modification strategies. The groups were offered at two different sessions each week, with flexible attendance for the participants who could switch between those times as needed. The groups met weekly via telehealth group video conferencing sessions for 16 weeks, and then every other week for 2 additional sessions in the final 4 weeks. Participants were provided a digital scale to track weight and access to MyFitnessPal.com for personal fitness and nutrition tracking.

Participants for the pilot GET-WEL Program were recruited from BCS who documented willingness to participate in an institutional weight loss program (NCT 04855552) in a prior survey performed by our research team [9]. One hundred twenty-two BCS completed a questionnaire regarding weight loss of which 70 BCS indicated a willingness to participate in the weight loss program. Twenty-one patients enrolled in the pilot Program.

### Data collection

The Mixed Methods Research Lab (MMRL) in the Department of Family Medicine and Community Health at Perelman School of Medicine was utilized to conduct all interviews and perform analyses. Prospective interviewees were identified as eligible by the study team and then were contacted by the MMRL using a combination of phone and email outreach. Informed consent was obtained from all individual participants included in the study. Interviews were conducted between June and August of 2023 using a semi-structured interview guide (Table 1), with data collection stopping once thematic saturation was reached. Compensation was commensurate to the length of the interviews. Non-participants of the GET-WEL Program were asked questions in a semi-structured telephone interview (Table 1) relating to reasons for not participating in the GET-WEL Program for which they received a $50 gift card. Participants of the GET-WEL Program were asked questions relating to facilitators and barriers of participating in the GET-WEL Program in a semi-structured interview (Table 1) for which they received a $100 gift card.

**Table 1:** Interview questions

Interview set for non-participant group	1. What is the most important reason you decided (not) to join the study? 2. Are there any other factors that influenced your decision? 3. Do you think you might have considered the study if a peer patient had contacted you first? 4. Would you have considered the study if your physician spoke with you about it? 5. Would you have considered the study if we provided a gift card?
Interview set for participant group	1. What do you like most about the program? 2. What do you like the least about the program? 3. Are there any things you would change about the program? 4. If you were to change the format, what would be the best format for you? - Group- based telehealth (keep it the same) - In-person group-based - Hybrid group, with some in person and some telehealth visits - A different time than what was offered (the previous group was at noon or 5 pm) 5. Were there any barriers that you had to overcome to attend the program? 6. What made it acceptable or easy to attend the program? 7. What makes accessing healthy foods hard for you? 8. What were some factors that helped you access healthy foods? 9. What makes maintaining steady exercise hard for you? 10. What factors helped you keep an exercise routine? 11. Have you continued with the healthy lifestyle beyond the 6-month program? If not, what barriers got in the way? If yes, what helped you continue?

### Data analysis

All interviews were transcribed verbatim, then were quality checked. All patient identifier information was removed by the MMRL. Two members of the MMRL (OBC and IAL) coded and analyzed the data using an integrated approach, which is an iterative process of determining theories of themes and patterns that are present in the data. The two coders co-coded 20% of the transcripts, meeting to resolve differences in coding to ensure intercoder reliability. Coding of the remaining transcripts was completed by a single coder (OBC) using the qualitative software NVivo. After coding, thematic reports overall and by each group were reviewed and compared.

## Results

### Interviewee characteristics

Eighty people were contacted to join the study, including 22 participants who indicated willingness to participate in a weight loss intervention at the initial survey period and 58 who did not. Twenty-four total qualitative interviews were conducted. Interviews were conducted amongst 9 GET-WEL participants (8 White and 1 Black BCS) and 15 GET-WEL non-participants (8 White, 6 Black, and 1 Asian BCS) to reach thematic saturation. The quotes that support the themes are illustrative of the sample as shown below.

### Perceptions and attitudes towards GET-WEL

When GET-WEL non-participants were asked why they did not participate in the GET-WEL Program, most non-participants cited that they were not aware that the Program was recruiting.

“*I don’t remember actually being asked, but I know that if I had been asked, I probably would have asked to be part of it [the study].*”

“*I filled out the paperwork but then I don’t remember getting additional information on whether to participate or not. I didn’t hear anything back and so I didn’t participate. I know I did, I agreed to participate, but I don’t remember receiving any additional information on how to participate.*”

Another non-participant mentioned they did not think the Program would provide them with adequate support or information to lose weight, as they perceived that the Program did not include access to a nutritionist or a similar specialized professional.

“*Well, it really wasn’t a weight loss study – there was no nutritionist involved or someone who was going to help me with that. So, my understanding of the project was more of a monitoring of what was in my life rather than a help. That to me with limited time in your life and with the focus, I was trying to focus away from cancer and not with a research study related to cancer.*”

One non-participant did not want to participate because they wanted to end their cancer journey.

“*I had cancer. I was moving toward being cancer free and I wanted to focus on taking care of myself and so a study that kept me in the cancer world, I didn’t want to be there. It was mental more than anything.*”

When GET-WEL participants were asked why they participated in the Program, most joined simply because they wanted to lose weight and believed the Program would help them achieve weight loss. They explained that losing weight was a way of taking care of themselves and “putting themselves first” after their cancer diagnosis and treatment.

“*It was a method of monitoring and structure and discipline and keeping track.*”

“*When I finally decided to lose weight, it was putting myself first… It was just time to put myself first. And I think when you have cancer, you’re just thinking about getting through the first month, the second month, the first year, the fifth year. And it was finally like I woke up one day and said, hopefully I have 25 good years left. I need to take care of myself so that I’m here and can do the things that I want to do.*”

When GET-WEL participants were asked how the Program could have been improved, they wanted to hear from more experts during the weekly meetings rather than from other BCS, which resulted in comparing themselves with other’s health status and did not result in any information they felt they could act on.

"*I think it would be helpful to have even a doctor, like a physician, present, just for guidance too. It’s like one of those breast cancer meetings that you go to and everybody has discussions and pitches in their information and their experiences. But to me, that’s all it is. You’re not really getting any hardcore information to help you and better your health and feel better physically and mentally, psychologically. I just think it would help if there were doctors involved rather than just the patients being involved.*"

Participants wished for more specific advice, especially when it came to enacting the nutritional guidance they were given. Specifically, they spoke extensively about feeling like they had too much contradictory information about nutrition and no longer felt they had clear guidelines to keep themselves healthy.

“*To actually know what [healthy foods] are. I mean, I know where to buy them. But the thing is, they say, ‘Oh, become a vegan. Oh, become a vegetarian. Oh, don’t eat meat. No red meats.’ But the vegetables are sprayed with all the toxins. We’re supposed to stay away from toxins. The meats have horrible effects on the body. Vegan. You have to actually know how to cook vegan in order to eat vegan even if you want to try it to be able to ingest it, because you have to acquire a taste for vegan. They say drink water. But then you have to worry about the plastics in the water bottles. And our water is poisoned and it’s always contaminated. Eat more fish. But then you do the studies on the fish and you find out that the fish are eating the microplastics that we are ingesting after we eat the fish. Eat more chicken. You eat the chicken and then you find out that these chickens are contaminated with bird flu or this flu or that flu. It’s insanity. You don’t know what to do.*”

### GET-WEL attendance—facilitators and barriers

All participants appreciated the flexibility, the telehealth format, and the ease of access to the sessions. The option for participants to attend either the noon or the 5 pm session via a virtual format made participation easier.

“*. . . the time of day I thought was good for me. I was working at the time so I think our appointment was at five o'clock. And that was good for me because it was after work. I didn't have to worry about taking time off or anything like that.*”

“*I think virtual makes it easier for everyone to make it to the meeting. I know a few times I had to go to a private room at work to be able to do it because I couldn’t get out of work in time. So, I think that virtual makes it a lot easier to get to the meeting.*”

Most participants reported that the Program afforded them accountability which was otherwise not available outside of a structured Program.

“*It kept me focused on diet, on exercise, on losing weight, which I desperately wanted to do anyway. I wanted to lose weight and so it really kept me focused and that was really very helpful.*”

### GET-WEL healthy lifestyle maintenance—facilitators and barriers

One of the more common themes relating to participants’ ability in maintaining their physical activity after completing the GET-WEL Program was whether they had found a physical activity they enjoyed doing. In addition, having a pre-existing exercise routine or habit was cited as another facilitator of physical activity.

“*I’m pretty active for my age. I’m out there on the pickleball courts maybe three days a week, I’m playing tennis one day a week, I’m walking when I’m not doing other things.*”

“*I was swimming two days a week and then three days a week on my cycle, whether it was indoors or outdoors and trying to get about, I think I averaged 150 minutes a week, if not more just because I like to work out.*”

“*[Being active is] not a new thing, it's lifelong, that really makes a difference. I mean, if you’ve been doing stuff all your life, practically certainly from adolescence forward, if not before. Then you know when you’re not doing enough.*”

Competing work/life priorities and time constraints was noted as a barrier in maintaining physical activity.

“*I am caretaking an ill relative and that person is 45 minutes away. So, when I go see him, that takes up my day and I tend not to exercise on those days.*”

Not finding an enjoyable physical activity was cited as another barrier.

“*. . . personally, I’m still trying to find what form of exercise I really enjoy. I’ve never been the person who like, I work out and work out and I hit that zone. I’m like, yes, this feels great. I would prefer to be a couch potato, but I do like walking my dog.*”

Other representative quotes are listed in Table 2.

**Table 2:** Additional representative quotes by theme

Interview theme		Representative quote(s)
Motivations for participation		“I did like that I lost weight. That was probably the best part of it. I also liked the ability to have other people's input, their suggestions, meeting with people. It was nice to have the Zoom and see them face-to-face.” (Interviewee 9)
Motivations against participation		“It'd been right away, you know, relatively short time after I had indicated interest, I might have gone ahead with it. I don't have anything against telehealth, it's just it took a long time and I had already made other arrangements.” (Interviewee 45) “[I didn’t join the study because] I always thought it was full.” (Interviewee 44)
Program results	Lifestyle changes maintained	“Yes, I have [maintained changes]. I feel as though I have. My weight has been pretty good and I've learned a lot as far as, like I said, eating habits and things and all that unnecessary snacking and things, I really kind of cut that out and I enjoy feeling better, have more energy and things like that, yes.” (Interviewee 67)
	Changes not maintained	“... it’s just once you lose the group, and you have no backup, you tend to slip. The group was, ‘Okay, we're going to meet again this week, and we're going to talk about our progress or where we have pitfalls.’ And things like that, and when you just lose that, it makes it difficult.” (Interviewee 53)
Program perceptions/attitudes and feedback	Pros	“The online guidance in the sort of lecture type format. I found that very helpful and very useful and informative…It was the format itself, the discussion, the content, the time limited approach, the kind of asking people to sort of review where they were and how things were going. I mean, it seemed to me it was a kind of relationship building approach and I thought it worked, at least it did for me.” (Interviewee 11) “The time of day were fine with me and I know with me being home and retired time was okay because I had both times were like, you know, it worked.” (Interviewee 67)
	Critique	“I think it would be helpful if there was more included on actual possible simple meals and there was nothing there as far as – it's obviously suggested eat this, don't eat this, but examples like different. Hey, here's good breakfast meals, here's good lunch meals, here's good dinner meals. Some suggestions of good things to eat. Like in a recipe form or not even a recipe form but which would be great too like breakfast. Hey, a piece of toast with avocado, and a slice of tomato. These kind of things to give suggestions of good ideas of what to put into your meal plans.” (Interviewee 53)

Regarding the maintenance of a healthy diet, GET-WEL participants reported having help with shopping (both guidance on what to buy, and someone who went to the grocery store for them) as a significant facilitator. Participants also said they were able to apply the dietary recommendations when they are eating or ordering out at restaurants.

“*When my daughter shops for me, it makes it easier. When I make up a list and I refer back to some of the things that they showed me on the program, I can do much better. Even when I go out to eat at a restaurant or something, my choices have gotten a little better because I'm knowledgeable about what would be better for me to eat and help with my nutrition.*”

Regarding the barrier to maintaining a healthy diet, participants discussed aspects of food access and preparation. They mentioned having to balance the cost and quality of their groceries.

“*Accessing healthy foods is harder, probably their availability . . . You have to shop at four different places sometimes to get what you need because no one grocery store seems to have the quality and the price that you might be looking for.*”

Participants said not cooking was a significant barrier to eating foods they deemed healthy. Participants who were not the primary cooks in their household reported having less control over their diet than the participants who prepared their own meals.

“*[My son] likes to cook and he always has my breakfast ready some days. He has meat and I don’t really. That’s hard for me, because they’ll tell you when we were doing the program, sometimes you just have to throw food away and if you're that type, you’re going to eat it even though it’s bad for you, because you're not going to waste it, so I am from that generation and I think that was hard and it still is.*”

Participants who did cook most of their own meals highlighted time constraints as a significant barrier to preparing balanced meals. They reported typically cooking themselves nutritious meals but not doing so when their work was in an especially busy period.

“*Busyness at certain times of the year – it’s just harder for me just because of my job and what I do. I lost about 25 pounds on this program and I was able to keep it off even through Christmas… And my springs are very busy. And so then I wasn’t eating as well and grabbing things at a restaurant, like going to Chipotle or whatever it is just not eating the same way. And I gained five pounds during probably May-June time.*”

Overall, 5 of the 9 participants reported they were maintaining the lifestyle changes they established during the Program while the other 4 were not able to maintain the lifestyle changes they adopted during the Program.

## Discussion

In this qualitative study, we sought to understand factors that may influence participation and non-participation in GET-WEL, as well as identify facilitators and barriers to implementation of the intervention that could be amplified or mitigated, respectively, in future studies. We found no discernable thematic differences between Black and White non-participants. The most common reason for non-participation was the lack of knowledge that the Program was open for enrollment which suggested that an enhanced Program information dissemination and outreach may increase Program awareness and mitigate non-participation, especially in minoritized BCS with obesity. For example, standardized emails, reminders at office visits, and phone calls to interested BCS may improve enrollment.

One of the GET-WEL Program facilitators that emerged from our interviews was related to how the GET-WEL Program was conducted. Other weight loss programs for BCS have been conducted via telephone [7, 13] or in person. For example, the multicenter BWEL study [13] utilized semi-structured telephone calls for weight loss intervention. However, GET-WEL’s format via telehealth video conferencing and flexible meeting times at noon or at 5 pm were noted to be convenient and allowed participants the flexibility to virtually attend the meeting at either time. The use of telehealth, especially during the COVID pandemic, has skyrocketed [14]. Patients have favorable views regarding telehealth due to its convenience and shorter wait times as compared to conventional in-person appointments [15]. Accordingly, telehealth has been shown to decrease appointment no-show rates overall and in particular for minoritized patients [16–18]. Thus, the telehealth format for delivering this behavioral weight loss Program was welcomed by nearly all GET-WEL participants. To our knowledge, this is the first weight loss program for BCS that uses telehealth video conferencing as the delivery method. However, the lack of opportunities for in-person socialization and building a sense of community with other participants remained a noted critique. Moving forward, a hybrid model may optimize engagement and allow for socializing and community building among those who prefer to attend in person.

Barriers to maintaining a healthy lifestyle included difficulty in accessing or affording healthy foods, and the lack of time and competing work/life priorities which limit the ability to prepare healthy meals and exercise. These barriers may be potentially modifiable and could be mitigated with resources provided by the Program or within the participants’ community. Socioeconomic status may also impact participation if potential participants do not have internet access for telehealth sessions.

Finally, the Program was led by a psychologist who was an expert in behavioral weight loss. Participants desired a wider range of experts, specifically dieticians, to lead sessions to gain further information specific to their survivorship experience. For example, dietitians who work with patients contribute to more patient weight loss on average than patients without a dietitian’s input [19]. Having social work expertise allows for resource distribution regarding affordable options for healthy foods, addressing the barrier of food expenses as noted by the participant group.

Limitations of this study included a small sample size and that all interviewees were recruited from a single institution. Participants in GET-WEL were limited to English-speaking patients due to the language limitation of the Program’s counseling sessions which inherently limits diversity in the participant population. For the purposes of this pilot and resource limitations, we only included English speakers, but we feel it is important to also make this intervention accessible for non-English speakers. We intend to do this in follow-up studies. In addition, interviewee responses were subjected to hindsight bias [20]. For example, non-participants may have been more likely to express in the interview their intent or interest in joining the Program if they had been asked previously, even if they would not have participated realistically at that time. Responses from the participant group may lean toward more positive comments as they may be subjected to the Hawthorne effect [21]. Only 24 of 80 contacted were interviewed, but thematic saturation was reached for both GET-WEL participants and non-participants.

In conclusion, the most common reason for GET-WEL non-participation was the lack of knowledge that the Program was open for enrollment, suggesting that an enhanced Program information dissemination and outreach may possibly mitigate the lower participation rate of minoritized BCS with obesity. Having resources to improve access to healthy foods and support to ease competing work/life priorities may help facilitate participants to continue to implement the intervention and maintain a healthy lifestyle after Program completion. Results of this qualitative study may help inform future large-scale GET-WEL Program recruitment and implementation.

## Data Availability

No datasets were generated or analysed during the current study.
